# Reply of Professor E. Nocard to Professor Koch

**Published:** 1902-01

**Authors:** 


THE JOURNAL

OF

COMPARATIVE MEDICINE AND

VETERINARY ARCHIVES.

VOL. XXIII. *JANUARY, 1902.* No. 1.

### REPLY OF PROFESSOR E. NOCARD TO PROFESSOR KOCH.[1]

I CONSIDER it a great honor to salute, in the name of French bacteriologists, the greatest bacteriologist of the whole world. To this tribute of admiration and respect I ask of this illustrious savant permission to make public acknowledgment of the gratitude which I expressed to him nearly ten years ago, on an occasion never to be forgotten, when I was able to judge of the elevation of his mind, the nobility of his character, and the rare goodness of his heart.[2]

A communication from Koch is always an event; that of to-day will go far and wide. Parts of it charm me and fill me with content; other parts trouble me—they appear to me full of danger.

I may be thought audacious to discuss the work of a master who is authority in all matters which concern bacteriology, and above all in regard to tuberculosis. I know that my intervention is somewhat dangerous. However, the interests at stake are so important, and the conclusions from Koch's work so fraught with grave consequences, that I cannot hesitate to respond to the invitation of our illustrious President, Lord Lister, to speak my sentiments on the subject.

That which pleases me in the communication of Koch is that he justifies in a clear manner the efforts of those who protest against

[1] Delivered in St. James' Hall, London, July 27, 1901, before the British Congress on Tuberculosis. Translated from "La Presse Médicale," July 27, 1901, by Mazyck P. Ravenel, M.D.

[2] Allusion is here made to the sympathetic devotion shown by Professor Koch to the French Commission, composed of Messrs. Roux, Strauss, Nocard, and Thuillier, sent to Egypt in 1883 to study the epidemic of cholera. It will be remembered that in forty-eight hours Thuillier was carried off by the disease.

182671

extreme methods in the prophylactic measures designed to protect the human race against the dangers of bovine tuberculosis. For some years a salutary reaction has taken place against the extreme measures at first adopted. The communication of Koch will accomplish this end. Perhaps it goes to extremes in the inverse sense, and I fear greatly that after having advised excessive and absurd measures against imaginary dangers people may now neglect to take precautions against the real dangers which exist for the public health in bovine tuberculosis.

I have always held—and recently again at the Berlin Congress on Tuberculosis—that bovine tuberculosis plays but a small part in the spread of human tuberculosis ; but no matter how small this part may be, it is undeniable, and it would be making a great mistake to leave it out of consideration.

Koch has not succeeded in producing tuberculosis in cattle inoculated by various methods with cultures or material from human sources. He concludes from this that cattle are refractory to human tuberculosis; that man has nothing to fear from tuberculosis of cattle, and that it is useless to take precautions against it.

With all the respect due to the illustrious savant, but with all the strength of profound conviction, I wish to say that his experiments do not justify such conclusions.

It is a principle of the experimental method that negative results, however great their number, cannot outweigh positive facts. There exist positive facts proving that it is possible to infect cattle with tuberculous matter from man. The first of these are the experiments of my eminent master, Professor Chauveau. I regret extremely that his duties prevented him from taking part in this Congress. He could have presented them with the authority which belongs to a master of experimental medicine. I will endeavor to take his place and to show the value of these facts from the point of view which now engages us.

Among his numerous experiments on this subject there are four which are particularly strong, in which calves aged from five to ten months were infected by the digestive tract, or by intravenous injection, with tuberculous material from man (acute phthisis or caseous pneumonia). These four animals were killed at different times up to fifty-nine days, and at the autopsy showed very important lesions, manifestly the result of the experimental infection.

In two of the three calves infected by ingestion the lesions were confluent in the intestine, the mesenteric glands, and on the peritoneum. In the third calf, on the contrary, while there were slight lesions of the abdominal organs, there existed very important ones in the glands related to the first part of the digestive tract, namely, the retropharyngeal and œsophageal glands.

Some will, no doubt, raise the objection to these experiments, that dating back to a period so far in the past when tuberculin was not known it was impossible to be sure that the calves used in the experiment were free from tuberculosis. Chauveau had foreseen this objection. He had chosen young calves for his experiments, because tuberculosis in calves is extremely rare. He procured these calves in a district where tuberculosis of cows was unknown, and finally in each experiment he preserved as controls an equal number of calves of the same age and origin, which were killed at the same time as the others, and were found to be absolutely free from tuberculosis.

Old as they may be, these experiments of Chauveau have on that account none the less value as positive facts against which negative experiments cannot prevail, however numerous they may be. Furthermore, several members of this Congress are announced to communicate analogous facts with the proofs.

These facts prove that although it may be difficult to communicate human tuberculosis to cattle, it nevertheless succeeds at times. How can we explain these different results, apparently contradictory? It is difficult to do this with certainty, for we cannot pretend that we are able to reproduce exactly in our experiments all the conditions of natural infection. It is, however, possible to give a plausible explanation. It is a well-known general law that the gradual adaptation of any parasite to a medium, inert or living, in which it can succeed in developing, confers on it the aptitude to develop more easily in media like the first. This is true for the bacillus of tuberculosis as for all other microbes. We know how difficult it is to obtain the first growth of the bacillus of Koch even on the most favorable culture medium. The first culture is always thin and scanty, but when once accustomed to our culture medium it grows afterward quite quickly and abundantly. What is true of inert media is still more true, *a fortiori*, of living media. Everyone knows that the bacillus of "rouget du porc" develops with difficulty at first in the organism of the rabbit. In order to kill one rabbit with certainty it is necessary to inoculate three or four animals, and death only occurs after four or five days, sometimes longer; but by passing this microbe from rabbit to rabbit it rapidly acquires such a degree of virulence that it kills rabbits in a few hours. But this bacillus, although it becomes so virulent for rabbits, has lost all its virulence for swine, from which it comes originally; we can inoculate swine with large doses without killing them and even without making them sick.

What I have said of the bacillus of "rouget du porc" applies also, and nearly as exactly, to the trypanosoma of dourine in the horse. This parasite of a higher order is inoculable into dogs, the white mouse, and the white rat among other animals. After some passages from mouse to mouse, or from rat to rat, it acquires such a degree of virulence that the animals die in a few days, showing an enormous number of trypanasomes in the blood. When, however, we inoculate the rat or mouse with this same trypanosoma after a certain number of passages through dogs, it no longer succeeds in killing them, nor does it make them sick. The prolonged adaptation of the trypanosoma to the organism of the dog causes it to lose its power of developing in the organism of the white mouse or white rat, but—a most curious thing—the trypanosoma of the dog remains always just as virulent for the horse.

Finally, I have shown that the tubercle bacillus from man or from cows cultivated in the peritoneal cavity of the chicken, protected from phagocytic action—thanks to the protection of a collodion sac—acquires, little by little, the characters of the avian tubercle bacillus, and becomes incapable of killing guinea-pigs, or kills them with lesions like those of avian tuberculosis.

All these facts lead me to think that the results obtained by Professor Koch proceed from causes of the same kind. Cattle rarely take tuberculosis from man; but when, for some reason or other, the resistance of the cells is modified, diminished, or suppressed, the human bacillus can multiply and invade the organs of the subject in which the resistance has been overcome. After this the bacillus, adapted to its new surroundings, is able to develop in other healthy cattle, which would show themselves refractory to the action of this same bacillus if it came directly from man. Let us admit, for an instant, that cattle are really refractory to human tuberculosis: has one the right to conclude from this that the reverse is equally true? No! a hundred times, no! This would be contrary to all the principles of the experimental method. It would be, above all, contrary to facts. If experimental facts are lacking, and for cause, clinical facts abound which prove the possibility of the transmissibility to man of tuberculosis of cattle. Many have seen veterinarians who have wounded themselves while making autopsies on tuberculous cows. Some have gotten well, thanks to prompt and radical surgical intervention, such as our colleague Jensen, of the Veterinary School of Copenhagen; others, less fortunate, have succumbed to the progressive evolution of the infection, such as our brethren Moses, of Weimar, and Thomas Walley, of the Royal Veterinary College of Edinburgh.

Furthermore, there exist numerous authentic instances of infection by the use of milk coming from cows with tuberculous mammitis. The best known and most authoritative concerns one of the daughters of Professor Gosse, of Geneva. It has almost the value of an experiment.

Finally, the work of the great English hygienist, Thorne-Thorne, proves as well as evidence the reality and gravity of the danger. During fifty years the mortality from tuberculosis in England has diminished 45 per cent.; during this same time abdominal tuberculosis of infants has increased 27 per cent. How can we explain these figures, so different? It is only for the last fifty years that you have done much in this country toward the cleansing of the house, the workshop, public places; lessening in this way the chances of infection by means of the respiratory tract, much the most common mode for adults; but you have done nothing against the dangers of infection by the digestive tract, which is the most frequent method for children artificially nourished.

Thorne-Thorne does not hesitate to attribute the increase of tuberculosis in young children to the absence of all regulations in regard to the dairy, and of all measures prohibiting the use of milk coming from cows with tuberculosis of the udder. All those who study this question of milk agree with the opinion of Thorne-Thorne. It is for these reasons that I will continue to teach to-day as yesterday—"Mothers of families, do not give milk to your babies unless the milk has been boiled."

---

Our State Veterinary Boards of Medical Examiners continue to grow in number, and each new board has some merit of advantage over its predecessors, and each new law adds some saving clause or feature over those which already exist. Gradually the work of those schools equipped to properly educate students for our profession is being defined by these laws, and the advance of the curriculums of the schools is a good omen of the future and a fitting tribute to those who have led and directed the work of establishing these safeguards to an unsuspecting public.

